# Unsupervised Hierarchical Clustering Identifies Immune Gene Subtypes in Gastric Cancer

**DOI:** 10.3389/fphar.2021.692454

**Published:** 2021-06-24

**Authors:** Jing Cao, Jiao Gong, Xinhua Li, Zhaoxia Hu, Yingjun Xu, Hong Shi, Danyang Li, Guangjian Liu, Yusheng Jie, Bo Hu, Yutian Chong

**Affiliations:** ^1^ Department of Infectious Diseases, Key Laboratory of Liver Disease of Guangdong Province, The Third Affiliated Hospital of Sun Yat-Sen University, Guangzhou, China; ^2^ Department of Laboratory Medicine, The Third Affiliated Hospital of Sun Yat-Sen University, Guangzhou, China; ^3^ Guangzhou Women and Children’s Medical Center, Guangzhou Medical University, Guangzhou, China

**Keywords:** gastric cancer, molecular subtypes, unsupervised hierarchical clustering, cancer classification, prognosis

## Abstract

**Objectives:** The pathogenesis of heterogeneity in gastric cancer (GC) is not clear and presents as a significant obstacle in providing effective drug treatment. We aimed to identify subtypes of GC and explore the underlying pathogenesis.

**Methods:** We collected two microarray datasets from GEO (GSE84433 and GSE84426), performed an unsupervised cluster analysis based on gene expression patterns, and identified related immune and stromal cells. Then, we explored the possible molecular mechanisms of each subtype by functional enrichment analysis and identified related hub genes.

**Results:** First, we identified three clusters of GC by unsupervised hierarchical clustering, with average silhouette width of 0.96, and also identified their related representative genes and immune cells. We validated our findings using dataset GSE84426. Subtypes associated with the highest mortality (subtype 2 in the training group and subtype C in the validation group) showed high expression of SPARC, COL3A1, and CCN. Both subtypes also showed high infiltration of fibroblasts, endothelial cells, hematopoietic stem cells, and a high stromal score. Furthermore, subtypes with the best prognosis (subtype 3 in the training group and subtype A in the validation group) showed high expression of FGL2, DLGAP1-AS5, and so on. Both subtypes also showed high infiltration of CD4^+^ T cells, CD8^+^ T cells, NK cells, pDC, macrophages, and CD4^+^ T effector memory cells.

**Conclusion:** We found that GC can be classified into three subtypes based on gene expression patterns and cell composition. Findings of this study help us better understand the tumor microenvironment and immune milieu associated with heterogeneity in GC and provide practical information to guide personalized treatment.

## Introduction

Gastric cancer (GC) is the fifth most common tumor and the third leading cause of cancer-associated death worldwide (Bray et al., 2018). Albeit unprecedented improvements achieved in endoscopic, surgical, and comprehensive treatments, the current global survival rates are poor (Arnold et al., 2020). Surgical resection with adjuvant chemoradiotherapy is the conventional treatment for resectable GC at advanced stages (Macdonald et al., 2001). The intricate immunosuppressive microenvironment, clinical heterogeneity, and molecular characteristics have been reported to contribute to resistance to current chemotherapy and/or radiotherapy plans (Bijlsma et al., 2017).

Molecular subtypes of GC have previously been developed based on gene expression profiling, mutation patterns, histopathological images, or gene sets such as DNA repair gene sets (Lei et al., 2013; Bass et al. 2014; Li X. et al., 2016; Liu et al., 2018; Jinjia et al., 2019). However, few studies have successfully integrated gene expression profiling with immune and stromal cell density to classify GC. Mounting evidence indicates that our immune system plays a crucial role during tumorigenesis and progression (Upadhyay et al., 2018), with much emphasis now being laid on immunotherapy. Most patients benefit little from immunotherapy, highlighting the need to investigate the pathogenesis behind GC heterogeneity and identify novel immunotherapeutic targets and prognostic markers in GC. Interestingly, recent studies have shown that stromal cells not only constitute an essential part of the tumor microenvironment but can also influence the phenotype of immune cells and tumor progression (Oya et al., 2020; Ren et al., 2020). A better understanding of the role of the stromal cell can help predict the prognosis and response to immunotherapy in GC patients.

In the current study, we provide substantial evidence that GC can be stratified into three clinically relevant subtypes with distinct survival patterns. We further identified each subtype’s representative genes as well as the immune and stromal cell composition. We successfully validated our findings using an independent dataset. In both the training and validation groups, subtypes with worst prognosis shared similar gene expression and cell composition pattern. Interestingly, our results emphasized the role of stromal cells and immune cells in explaining GC interpatient heterogeneity.

## Method

### Patients and Gene Expression Microarray Data Acquisition

The National Center for Biotechnology Information (NCBI) Gene Expression Omnibus (GEO, http://www.ncbi.nlm.nih.gov/geo) database is a public functional genomics database with high throughput gene expression sequencing data and microarray data. Gene expression profile of GSE84433 based on GPL6947 platform was downloaded and consisted of 357 tissue samples of GC. GSE84426, another dataset, which consisted of 76 tissue samples based on the same platform (GPL6947), was used for validation.

### Unsupervised Cluster Analysis

The R package CancerSubtypes Xu et al. (2017) was used for identifying, validating, and visualizing molecular cancer subtypes from multi-omics data. The algorithm for feature selection, based on a multivariate Cox regression model (features included gene expression, overall survival time, status, and cutoff <0.05), was applied on GSE84433 dataset. Then, the clustering method, consensus nonnegative matrix factorization (NMF), was used to identify different subtypes. NMF is an unsupervised learning method for pattern recognition on gene expression profiling and cell composition used to classify genes into clusters. The default number of runs was set to 30 to allow computation of a consensus matrix for selection of the best possible results. The silhouette width index was calculated to assess accuracy and fitness of the clustering assignment. Silhouette width values vary between −1 and 1. The more it tends to approach 1, the better the degree of cohesion and separation. The xCell function of “Immunedeconv” Sturm et al. (2020), another R package, was used for analysis of tumor-infiltrating immune cells. xCell can show the relative enrichment of predetermined combinations of gene profiles and performs cell-type enrichment analysis from gene expression data for 64 immune and stromal cell types. The same algorithm for subtype identification as mentioned above was used for immune cell-type identification. The “ComplexHeatmap” package was then used to draw heatmaps. We compared patient survival rates among these clusters using survival analysis in cancer subtypes. *p* < 0.05 represented statistically significant difference.

### Analysis of Correlation Between Different Clusters and Clinical Features

The “Tableone” package in R was used for analysis of clinical characteristics of the three GC clusters (subtypes). *p* < 0.05 represented statistically significant difference. “ComplexHeatmap” package was used to generate heatmaps.

### Analysis of Differences Among Different Subtypes

We utilized the “limma” package to identify differentially expressed genes in each subtype (the threshold value for differentially expressed genes (DEGs) was set at *p* < 0.05 and the absolute value of log two fold change (FC) > 1.8). The representative DEGs were identified by a Venn diagram method. For example, to identify the DEGs of subtype 1, a Venn diagram of (subtype 1 + subtype 2) v/s (subtype 1 + subtype 3) was drawn to obtain the overlap of DEGs. With a threshold value of *p* < 0.05 or 1.5-fold, screening of immune cells for each subtype was performed, followed by cross analysis, to obtain infiltrated immune cells of each subtype. “ComplexHeatmap package” was used to generate heatmaps.

Last, cell types were also predicted using xCell analysis. The score for each cell type on a heatmap explicitly shows the enrichment of the certain cell type compared to other regions within the same section.

### Function and Pathway Enrichment Analyses

To understand the biological characteristics of each subtype, we applied a functional enrichment test to differentially expressed genes of each subtype. “clusterprofiler” were applied to investigate the molecular function (MF), while “ReactomePA” was applied for analysis of the reactome pathway.

### Construction of Protein–Protein Interaction Network and Analysis of Hub Genes

Protein–protein interaction (PPI) network analysis is a powerful tool that can be harnessed to better understand the biological responses occurring in each gastric cancer subtypes. In the PPI network, a protein is defined as a node, and the interaction between two nodes is defined as an edge. The size of a node represents a degree: the larger the node, the larger the degree. The thickness of an edge indicates a correlation: the thicker the edge, the higher the correlation (Kohl et al., 2011). The online database STRING (https://string-db.org/) was applied to construct a PPI network of the genes and analyze the functional interactions between proteins. A confidence score ≥0.400 was set as significant. Cytoscape was then used to analyze hub genes, which are important nodes with many interactions, and visualize the PPI networks. Cytoscape plug-in molecular complex detection (MCODE) was used to screen the modules of the PPI network identified. The default settings of MCODE were set with a degree cutoff at 2, node score cutoff at 0.2, K core at 2, and a maximum depth of 100. The topological algorithm “degree” was applied in Cytoscape plug-in CytoHubba to calculate the importance of these hub genes in the PPI network (Chin et al., 2014).

### Validation Set

To verify reproducibility of our findings and investigate the relationship between differentially expressed genes and immune cells with the prognosis of GC patients, the GSE84426 dataset was downloaded from the GEO database. The CancerSubtypes package was used with feature selection based on the Cox regression model. Then, the NMF package was applied to identify clusters. Parameters were set for the training group (see method *Unsupervised Cluster Analysis*). The silhouette width was calculated to determine the accuracy of clustering assignment. The xCell function of “Immunedeconv,” another R package, was used for analysis of tumor-infiltrating cells. Differentially expressed genes and immune cells of all clusters were then compared.

## Results

### Identification of Three Subtypes by Unsupervised Hierarchical Cluster Analysis

**FIGURE 1:**
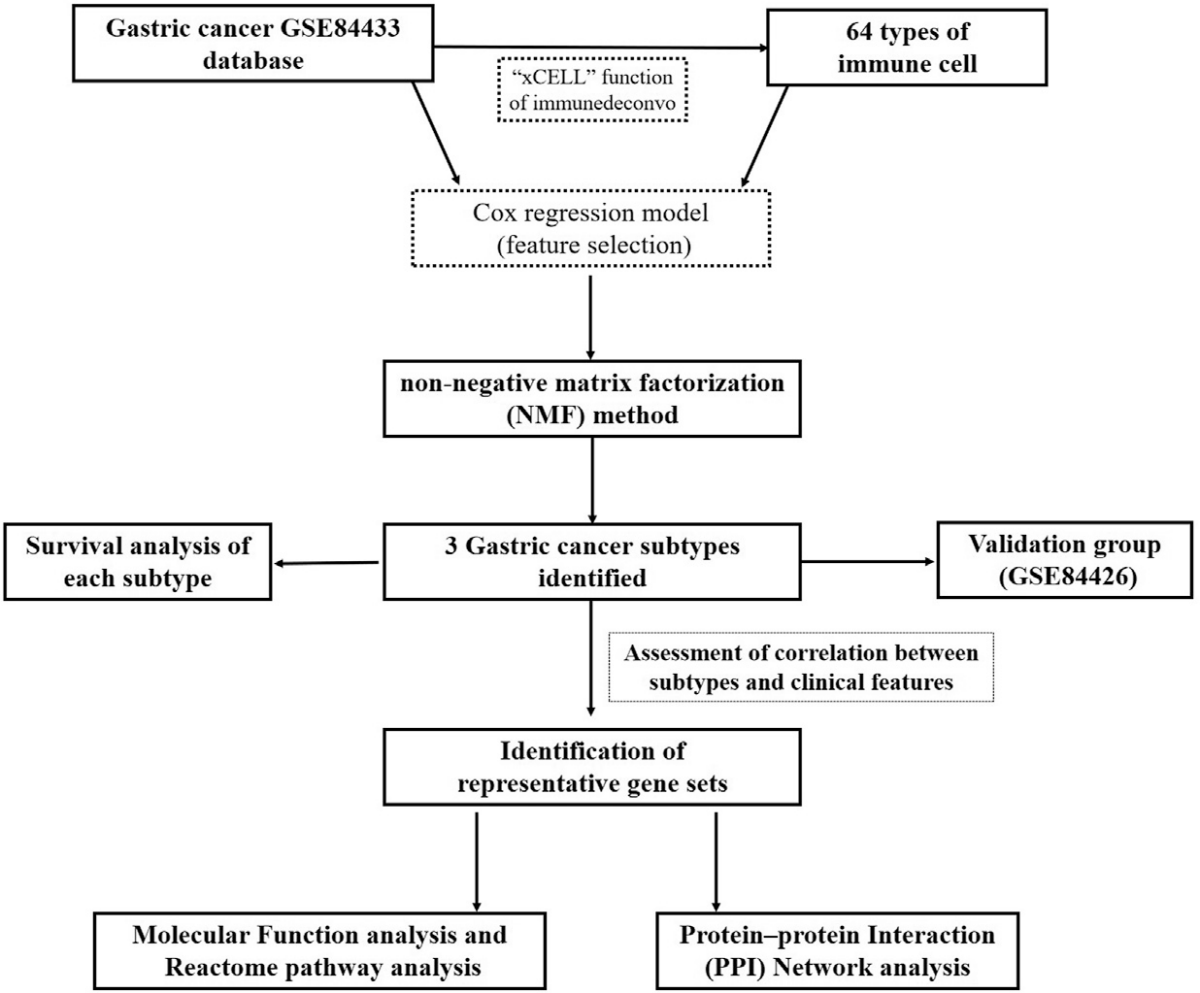
Flowchart of this study.

A flowchart detailing the overall study is shown in Figure 1. In order to assess tissue heterogeneity and to predict cell patterns, we first utilized the xCell algorithm and then unsupervised hierarchical analysis on the GSE84433 dataset.

**FIGURE 2:**
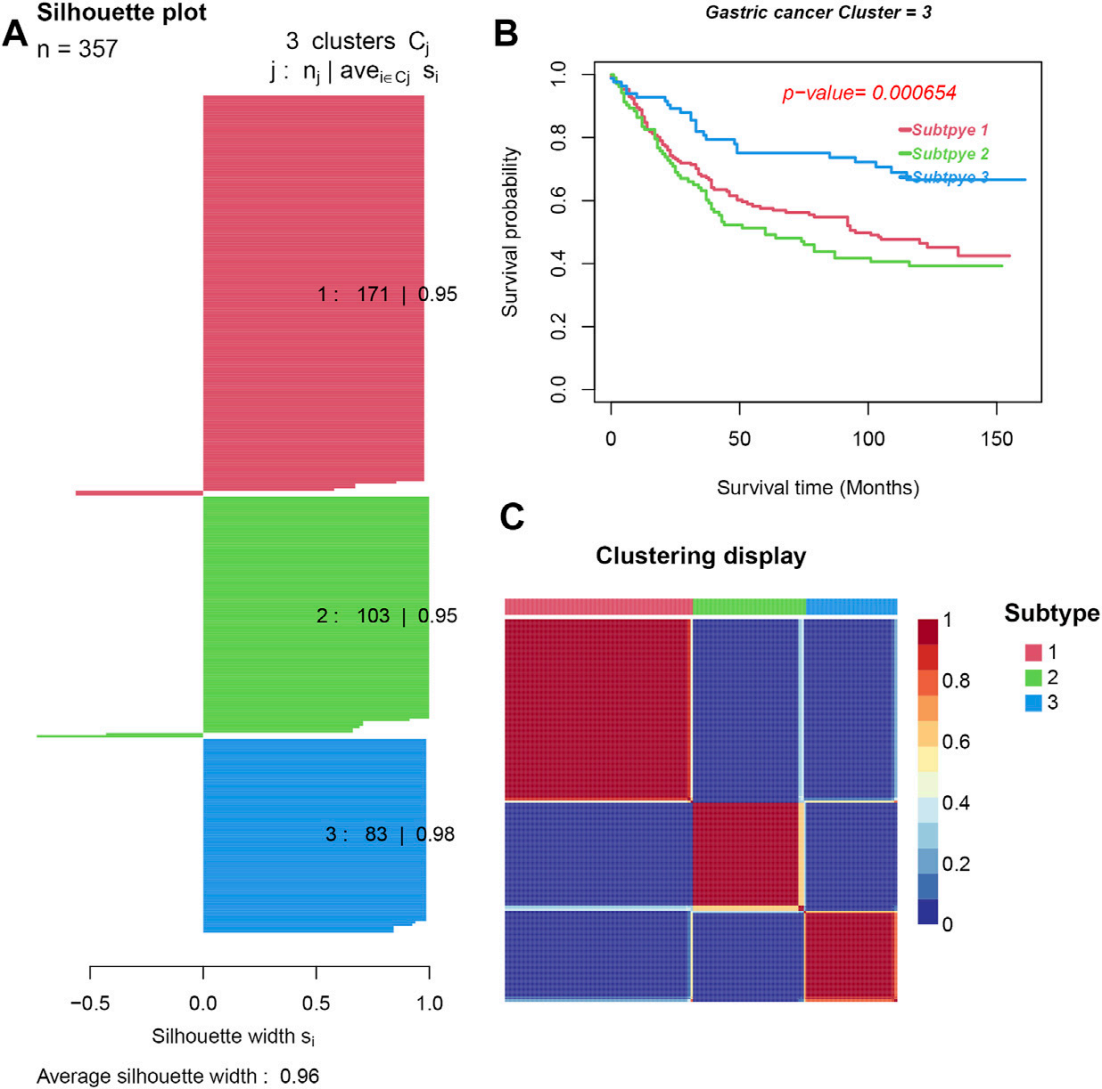
Three subtypes were obtained by unsupervised learning. **(A)**. Silhouette plots for the identified cancer subtypes. **(B)** Survival curves. **(C)**. Heatmap of the sample similarity matrix.

Feature selection based on a multivariate Cox regression model resulted into 2,358 significant genes and 32 significant immune cells. Three distinct GC subtypes were then identified by the NMF method. The silhouette width (used to determine the accuracy of clustering assignment) of subtype 1, subtype 2, and subtype 3 was 0.95, 0.95, and 0.98, respectively, while the average silhouette width was 0.96 (Figure 2A). The differential genes expression and density of cells in the three subtypes are shown in Figure 2C. Survival analysis subsequently showed that subtype 3 had the best prognosis, while subtype 2 had the worst prognosis (*p* = 0.000654) (see Figure 2B).

### Correlation Between Clinical Characteristics and GC Subtypes

**FIGURE 3:**
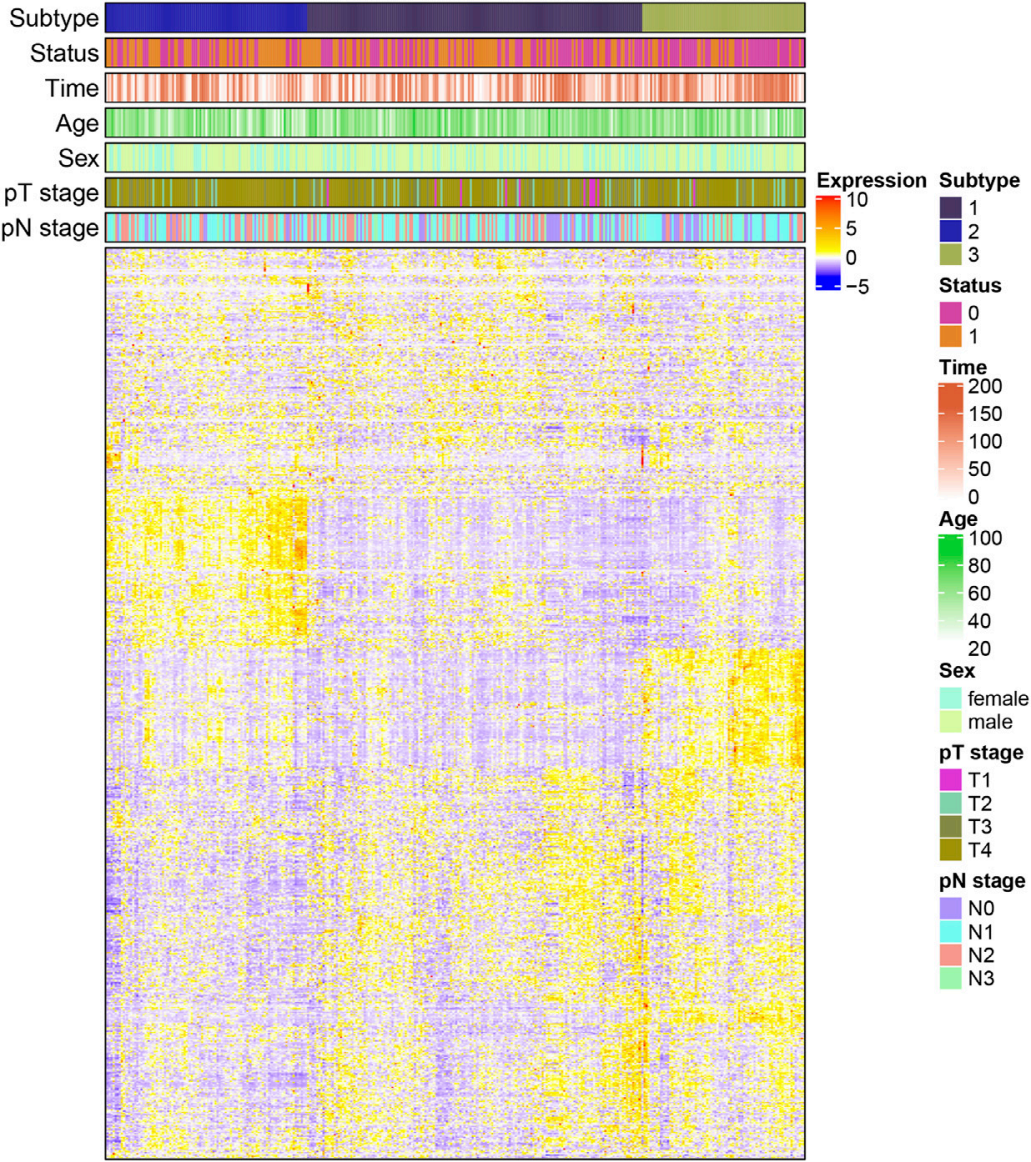
Heatmap of the correlation between differential genes expression and clinical characteristics in each subtype (pT: pathological primary tumor, pN: pathological lymph node status).

Statistical analysis of the difference in clinical characteristics (survival status, overall survival time, age, gender, pathological tumor (pT) stage, and pathological node (pN) stage) among the three subtypes was performed. DEGs in each subtype were then obtained. As seen in Figure 3, there was a significant difference of DEGs among all three subtypes, especially between subtypes 2 and 3. The intercluster comparison results are displayed in Table 1. Subtype 3 had the lowest mortality rate (30.1%), with a mean overall survival time (87.33 months), followed by subtype 1 which had a mortality rate of 51.5% and a mean survival of 71.16 months. Subtype 2 had the highest mortality rate (59.2%) and shortest mean survival (66.89 months).

**TABLE 1:** Analysis of the clinical characteristics of the three subtypes from the training dataset.

Variables	1 (*n* = 171)	2 (*n* = 103)	3 (*n* = 83)	*P* value
Status				
Death (%)	88 (51.5)	61 (59.2)	25 (30.1)	<0.001
Overall survival time (mean; SD) (month)	71.16 (49.60)	66.89 (50.05)	87.33 (47.65)	0.013
Age (mean; SD) (years)	61.37 (10.23)	56.88 (12.44)	58.95 (11.39)	0.005
Sex = male (%)	119 (69.6)	66 (64.1)	57 (68.7)	0.627
pT stage (%)				0.063
T1	10 (5.8)	0 (0.0)	1 (1.2)	
T2	18 (10.5)	8 (7.8)	9 (10.8)	
T3	36 (21.1)	16 (15.5)	15 (18.1)	
T4	107 (62.6)	79 (76.7)	58 (69.9)	
pN stage (%)				0.062
N0	37 (21.6)	15 (14.6)	19 (22.9)	
N1	66 (38.6)	45 (43.7)	44 (53.0)	
N2	49 (28.7)	36 (35.0)	14 (16.9)	
N3	19 (11.1)	7 (6.8)	6 (7.2)	

pT, pathological primary tumor; pN: pathological lymph node status.

**FIGURE 4:**
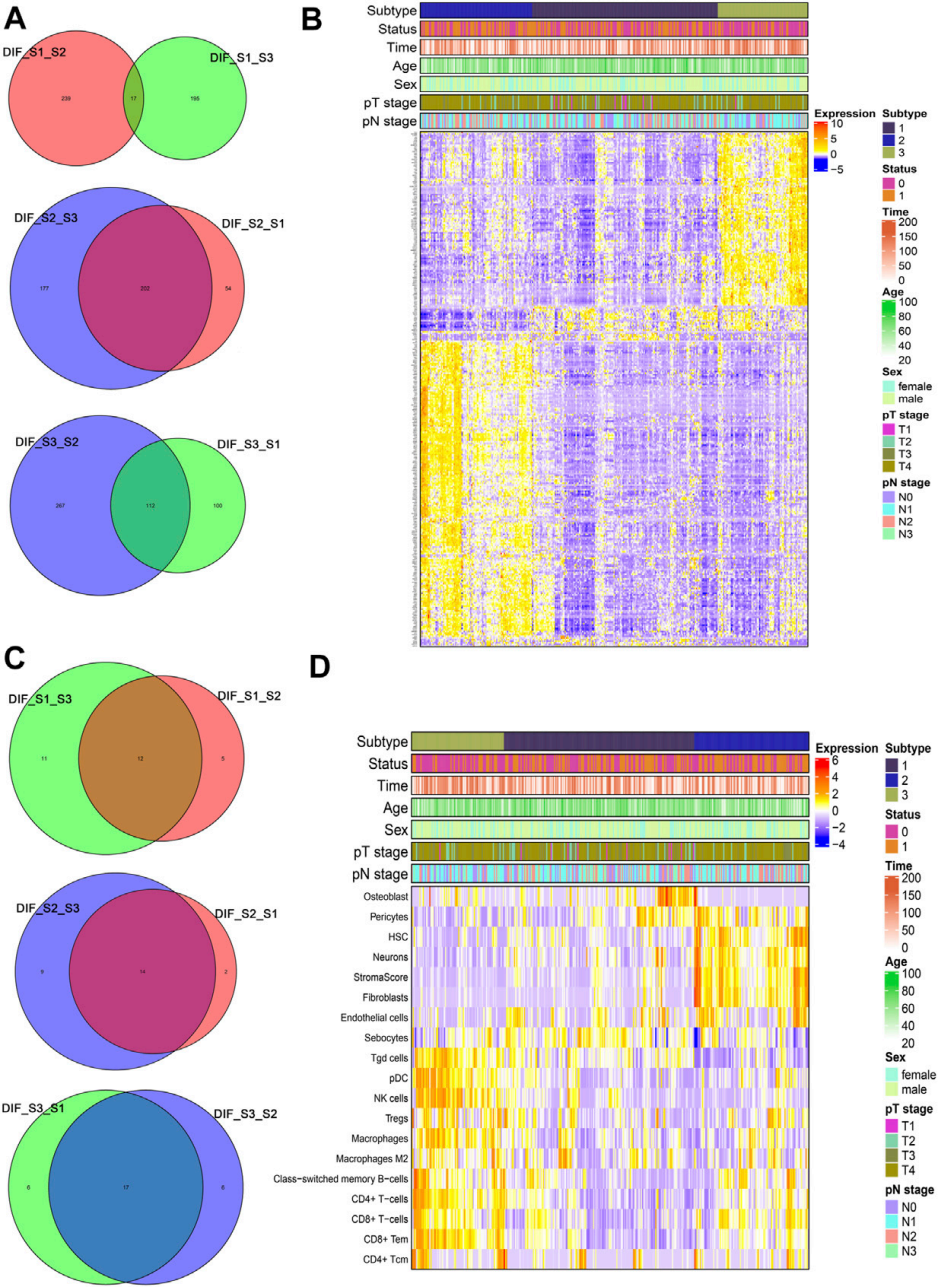
Representative differential genes and immune cells among different subtypes **(A and C)** Venn diagram; **(B and D)** heatmap.

To identify representative genes of each subtype, a Venn diagram method was used (Figure 4A) and expressed as a heatmap (Figure 4B). Subtype 2 displayed elevated expression of AKAP12, CPE, SSPN, TMEM47, GASK1B, ZNF521, CDO1, MIR99AHG, THBS4, LPAR1, CCN3, and so on. Subtype 3 showed high expression of FGL2, DLGAP1-AS5, and so on (see Supplementary Table S1 for complete list). There was a statistically significant difference, especially between subtypes 2 and 3 (Figure 4B). Moreover, to identify representative cells of each cluster, a Venn diagram method was used (Figure 4C) and expressed as a heatmap (Figure 4D). As seen in the heatmap, subtype 2, which had the worst prognosis, showed high expression in hematopoietic stem cells (HSCs), fibroblasts, and endothelial cells, and a high stroma score, while subtype 3 with the best prognosis consisted of CD4^+^ T cells, CD8^+^ T cells, NK cells, pDC, macrophages, CD4^+^ T effector memory cells, and Th1 cells (see Supplementary Table S2 for complete list).

### Pathway Analysis of Differentially Expressed Genes of Each Subtype

**FIGURE 5:**
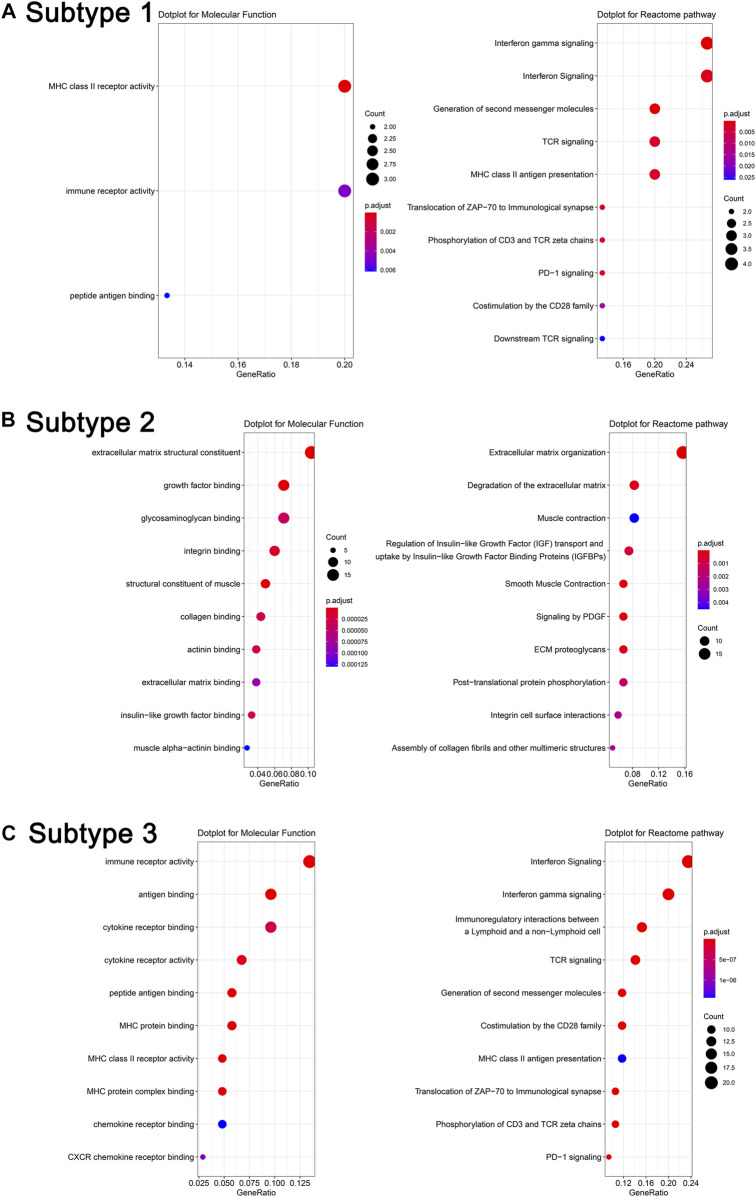
Molecular function analysis and reactome pathway analysis of representative differentially expressed genes in different subtypes **(A)**. Subtype 1, **(B)**. Subtype 2, and **(C)**. Subtype 3.

**FIGURE 6:**
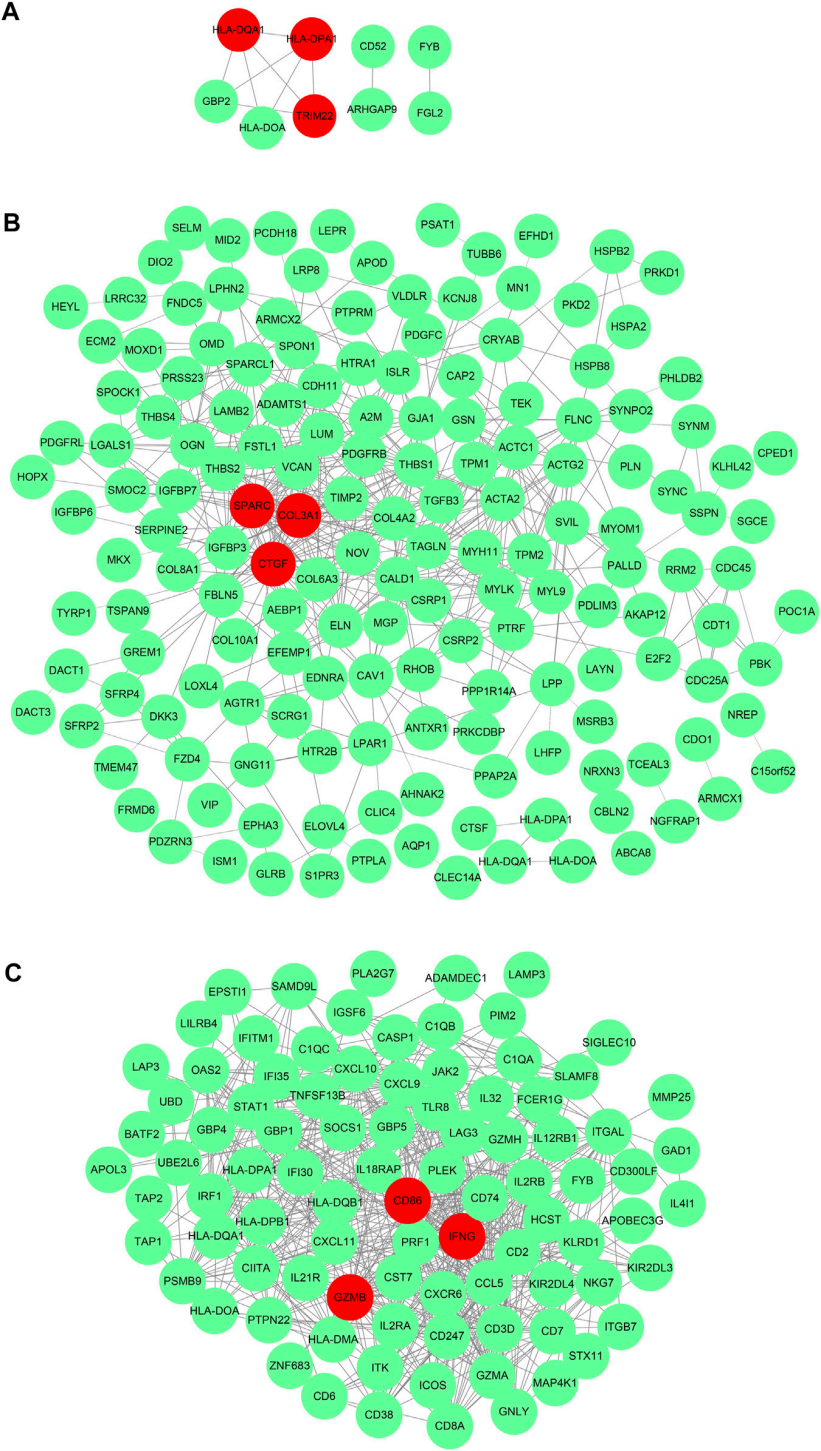
_Protein–protein interaction network of representative differential genes in each subtype **(A)**. Subtype 1, **(B)**. Subtype 2, and **(C)**. Subtype 3.

After obtaining DEGs of each subtype, we sought to understand the biological characteristics related to each subtype; we thus applied Gene Ontology (GO) enrichment analysis and reactome pathway analysis. As seen in Figures 5A–C, the MF of subtype 1 was characterized by “MHC class II receptor activity and immune receptor activity,” and the reactome pathway by “*γ*-interferon signaling, generation of second messenger molecules, and TCR signaling.” Subtype 2 was characterized by “Extracellular matrix structural constituent, growth factor binding, glycosaminoglycan binding, and integrin binding,” while reactome pathway processes included “degradation of the extracellular matrix, regulation of insulin-like growth factor transport and uptake by insulin-like growth factor binding proteins, and signaling by platelet-derived growth factor (PDGF).” Subtype 3 was characterized by “immune receptor activity, antigen binding, cytokine receptor activity, and binding,” while reactome pathway processes included “γ-interferon signaling, immunoregulatory interactions between a lymphoid and a nonlymphoid cell, and TCR signaling.” The PPI network of each subtype is displayed in Figures 6A–C. Hub gene proteins for subtype 1 included “HLA-DQA1, HLA-DPA1, and TRIM22”; subtype 2 included “SPARC (secreted protein, acidic, and rich in cysteine), COL3A1, and CTGF”; and subtype 3 included “CD86, interferon-gamma (IFNG), and granzyme B (GZMB).”

### Differential Genes and Immune Cell Analysis of Validation Set

**FIGURE 7:**
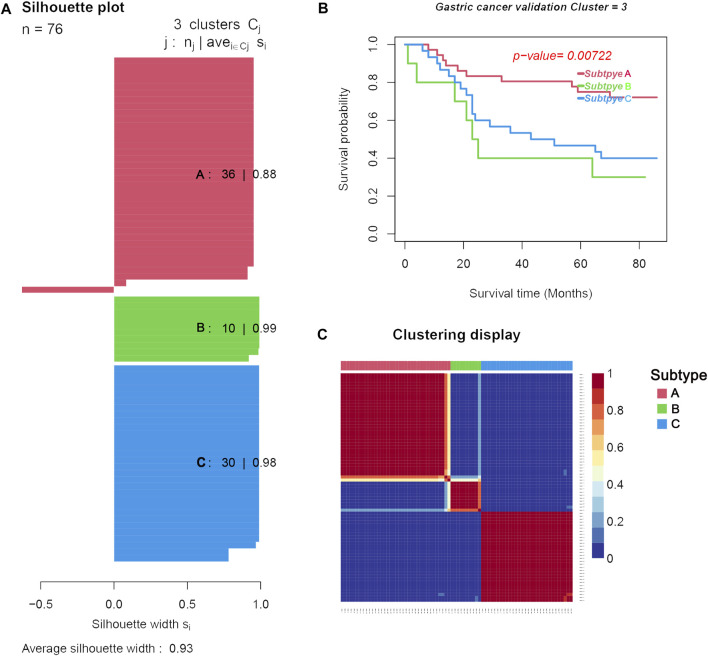
Three distinct subtypes obtained from the validation set (GSE84426) based on differential immune cell and genes. **(A)**. Silhouette plots for the identified cancer subtypes. **(B)**. Survival curves. **(C)**. Heatmap of the sample similarity matrix.

Another microarray dataset (GSE84426) consisting of 76 cases of GC was downloaded from the GEO database. The dataset was clustered into three subtypes (validation group subtypes A, B, and C) by NMF. As shown in Figure 7A, the silhouette widths of subtype A, subtype B, and subtype C were 0.88, 0.99, and 0.98, respectively, and the average silhouette width = 0.93. Survival analysis showed that the prognosis of subtype A in the validation set was significantly better than that in the other two subtypes (*p* = 0.00722; in Figure 7B). Results of the analysis of differential gene expression and immune cells in each subtype are shown in Figure 7C.

We compared the difference in clinical characteristics among the different subtypes of GC (Table 2). Subtype A had the lowest mortality (0.45%), followed by subtype B (0.48%), and subtype C had the highest mortality (0.50%). Subtype A had the longest overall survival time (64.06 months) compared to subtype C which was 46.53 months, while subtype B had the shortest overall survival time (39.10 months).

**TABLE 2:** Analysis of the clinical characteristics of the three subtypes from the validation dataset.

Variables	A (*n* = 36)	B (*n* = 10)	C (*n* = 30)	*P* value
Status				
Death (%)	0.28 (0.45)	0.70 (0.48)	0.60 (0.50)	0.008
Overall survival time (mean; SD) (month)	64.06 (24.57)	39.10 (32.13)	46.53 (27.88)	0.008
Age (mean; SD) (years)	65.64 (9.09)	64.70 (15.17)	58.33 (14.19)	0.050
Sex = male (%)	26 (72.2)	9 (90.0)	19 (63.3)	0.267
pT stage (%)				0.017
T1	0	0	0	
T2	3 (8.3)	0 (0.0)	0 (0.0)	
T3	17 (47.2)	3 (30.0)	5 (16.7)	
T4	16 (44.4)	7 (70.0)	25 (83.3)	
pN stage (%)				0.566
N0	4 (11.1)	0 (0.0)	5 (16.7)	
N1	18 (50.0)	5 (50.0)	10 (33.3)	
N2	14 (38.9)	5 (50.0)	14 (46.7)	
N3	0 (0.0)	0 (0.0)	1 (3.3)	

pT, pathological primary tumor; pN: pathological lymph node status.

We then analyzed the specific differential genes and differential immune cells among the three subtypes, as shown in Supplementary Figures S1, S2, respectively. Interestingly, subtype C with the highest mortality (50%) showed high expression of FOXF2, CAP2, MYLK, FLNC, AKAP12, CPE, SSPN, TMEM47, GASK1B, ZNF521, CDO1, MIR99AHG, THBS4, LPAR1, CCN3, and so on, with x-Cell results showing high infiltration of fibroblasts, endothelial cells, and HSCs, and a high stromal score. These results were similar to those obtained for subtype 2 in the training group.

## Discussion

The composition of immune and stromal cells in the tumor microenvironment contributes to tumor heterogeneity, and plays an essential role in determining treatment efficacy and patient prognosis (Mcgranahan and Swanton, 2017; Stein et al., 2019). Gene expression profiling using microarrays or RNA sequencing (RNA-seq) has been widely used to generate a wealth of transcriptomic profiles in many cancer types. In our study, we utilized xCell for cell-type enrichment analysis and performed unsupervised hierarchical analysis for pattern recognition on gene expression and cell composition. The correlation between GC subtypes and clinical characteristics was comprehensively assessed.

We found that GC could be classified into three clinically relevant subtypes with distinct survival patterns. The gene expression pattern and cell composition of two independent GC datasets, each divided into three subtypes by unsupervised cluster analysis, were similar. Subtypes associated with the highest mortality (subtype 2 in the training group and subtype C in the validation group) showed high expression of AKAP12, CPE, SSPN, TMEM47, GASK1B, ZNF521, CDO1, MIR99AHG, THBS4, and so on. Both subtypes also showed high infiltration of fibroblasts, endothelial cells, and HSCs, and a high stromal score. Subtypes with the best prognosis (subtype 3 in the training group and subtype A in the validation group) showed high infiltration of CD4^+^ T cells, CD8^+^ T cells, NK cells, pDC, macrophages, and CD4^+^ T effector memory cells. It has been reported that in GC, stromal cell types such as fibroblasts, endothelial cells, and HSCs lead to poor prognosis (Mao et al., 2020). Our findings were consistent with those of the previous study.

PPI network analysis showed the subtype 3 with the best prognosis was associated with hub genes “CD86, IFNG, and GZMB,” while functional enrichment tests showed MF involvement with biological processes such as “immune receptor activity, antigen binding, cytokine receptor activity, and binding,” terms related to cytotoxic antitumor immunity such as “*γ*-interferon signaling, immunoregulatory interactions between a lymphoid and a non-lymphoid cell, and TCR signaling” were found to be significantly enriched. Interestingly, the list of differentially expressed genes obtained for subtype 3 also included genes coding for interferon-*γ*, tryptophanyl-tRNA synthetase (WARS), CD8A, and guanylate-binding protein 4 (GBP4). In a retrospective study, patients with granzyme B and WARS had an improved 5-year overall survival with adjuvant chemotherapy on resectable GC (Cheong et al., 2018). Interferon-gamma (IFN*γ*) can enhance cytotoxic activities of natural killer cells and cytotoxic T lymphocytes (CTL), making tumor cells more prone to recognition and destruction. Many immunotherapeutic drugs including CTLA-4 and PD-1 inhibitors eliminate cancer cells by increasing IFN*γ* expression. Hurkmanns et al. found that lower granzyme B levels in patients with metastatic cancer favored tumor growth by halting the antitumor immunity response by cytotoxic immune cells (Hurkmans et al., 2020). Co-stimulatory molecules such as CD86 which are expressed on mature DCs are critical in the activation of naïve T cells (Hubo et al., 2013). These may explain why interferon-*γ*, granzyme B, and CD86 were associated with the best prognosis subtype.

Moreover, subtype 2 with the worst prognosis was associated with “SPARC, COL3A1, and CTGF,” and functional enrichment tests showed MF involvement with “extracellular matrix structural constituent, growth factor binding, glycosaminoglycan binding, and integrin binding,” while reactome pathway processes included “degradation of the extracellular matrix, regulation of insulin-like growth factor transport and uptake by insulin-like growth factor binding proteins, and signaling by PDGF.” Interestingly, Li Z. et al. (2016) reported that high immunostaining of SPARC, a matricellular glycoprotein, correlated with tumor differentiation and independently predicted shorter overall survival in 137 GC cases. Cheng et al. (2014) reported Cyr61/CTGF/Nov (CCN) proteins are part of a family of matricellular proteins that participate in GC carcinogenesis. Prior studies also revealed high CCN2 expression in GC correlated with a greater number of lymph node metastases, peritoneal dissemination, and shorter survival (Liu et al., 2007; Liu et al., 2008; Jiang et al., 2011).

In our study, differences in the cellular infiltrates among all subtypes were also established using xCell. Subtype 3 which showed the best prognosis featured the greatest number of cytotoxic antitumor immunity–related cell types. Both subtypes associated with the highest mortality (subtype C in the validation group and cluster two in the training group) showed high infiltration of fibroblasts, endothelial cells, and HSCs, and a high stromal score. This finding is in line with that of the previous study by Min *et al.* (Mao et al., 2020), which concluded that a high stromal score was a poor prognostic factor in GC. These clearly emphasize the role of cellular immunity and stroma in explaining the different prognosis of GC patients. Poor prognosis was associated with the presence of fibroblasts, endothelial cells, and HSCs.

One limitation of this study is our inability to correlate our findings with subtypes of the Lauren histological classification (Lauren, 1965). No histological data were available from the downloaded GSE84433 dataset. According to the Lauren classification, gastric adenocarcinoma can be classified into diffuse, intestinal type, and mixed type. The diffuse subtype is associated with aggressive progression, peritoneal metastasis, and poorer prognosis than intestinal-type GC. A recent study by Jinawath et al. discovered that SPARC which is involved in the production of extracellular matrix was enhanced in diffuse-type, and not in intestinal-type GC (Jinawath et al., 2004). Similarly, enhanced expression of genes usually involved in the production of ECM components in diffuse-type GC, including SPARC and COL3A1, was found in the worst prognosis subtype in our study. Moreover, results of our analysis are preliminary and need to be further validated at the clinical level. At present, biomarkers are needed to assist clinicians in drug selection and avoid toxicity in nonresponsive patients. Proper integration of gene expression profiling can help tailor disease management and improve the accuracy of current palliative chemotherapeutic measures.

## Conclusion

In a nutshell, based on unsupervised learning and xCell, we successfully stratified GC into three clinically relevant subtypes with distinct survival patterns. Importantly, we identified each subtype’s representative genes and immune and stromal cell composition. Interestingly, our results emphasized the role of stromal cells and immune cells in determining patient prognosis.

## Data Availability

The datasets presented in this study can be found in online repositories. The names of the repository/repositories and accession number(s) can be found in the article/Supplementary Material.
