# Supplementary figures and images for: Unsupervised Hierarchical Clustering Identifies Immune Gene Subtypes in Gastric Cancer
Source: Front Pharmacol. 2021 Jun 24;12:692454. doi: 10.3389/fphar.2021.692454 (PMC8264374; doi:10.3389/fphar.2021.692454)

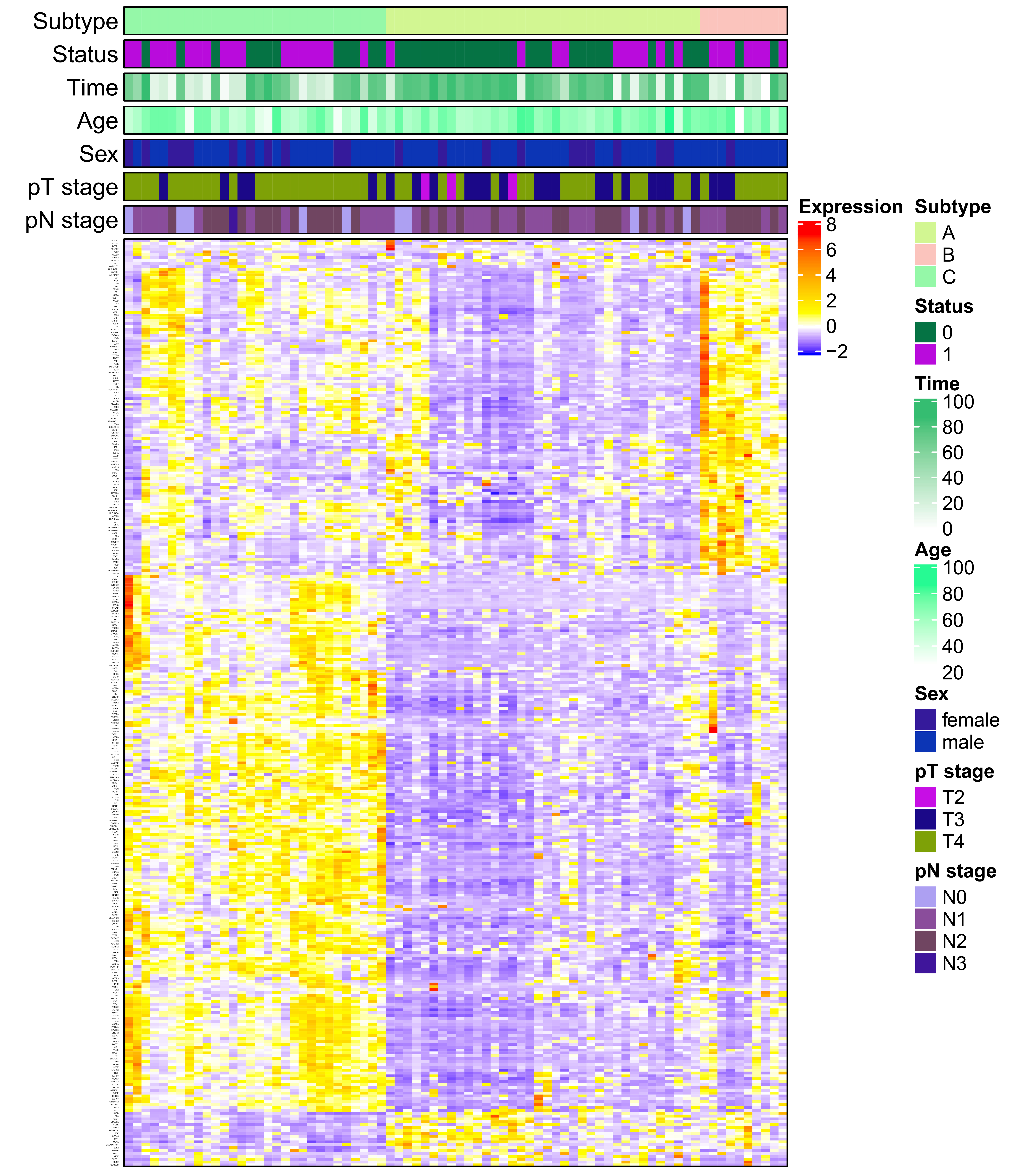

Supplement: Supplementary file 3 [file Image1.JPEG]

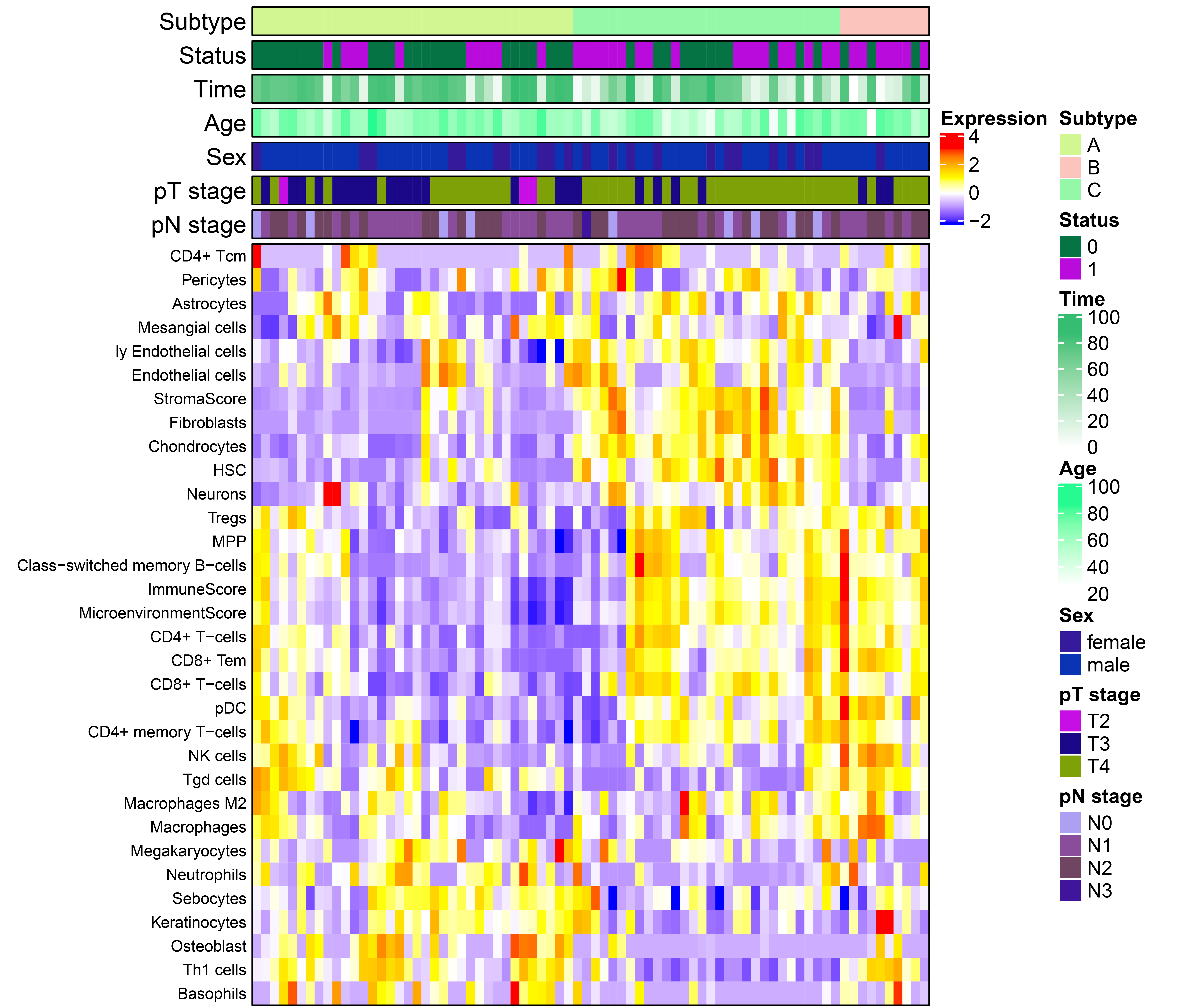

Supplement: Supplementary file 4 [file Image2.TIF]
